# Effects of age, comorbidity and adherence to current antimicrobial guidelines on mortality in hospitalized elderly patients with community-acquired pneumonia

**DOI:** 10.1186/s12879-018-3098-5

**Published:** 2018-04-24

**Authors:** Xiudi Han, Fei Zhou, Hui Li, Xiqian Xing, Liang Chen, Yimin Wang, Chunxiao Zhang, Xuedong Liu, Lijun Suo, Jinxiang Wang, Guohua Yu, Guangqiang Wang, Xuexin Yao, Hongxia Yu, Lei Wang, Meng Liu, Chunxue Xue, Bo Liu, Xiaoli Zhu, Yanli Li, Ying Xiao, Xiaojing Cui, Lijuan Li, Jay E. Purdy, Bin Cao, for the CAP-China network

**Affiliations:** 1 Department of Pulmonary and Critical Care Medicine, Beijing Chao-Yang Hospital, Capital Medical University, Gongti South Road, Chao-yang District, Beijing, China; 2 Department of Respiratory Medicine, Qingdao Municipal Hospital Group, Jiaozhou Road, Qingdao City, Shandong Province China; 3 National Clinical Research Center of Respiratory Diseases, Center for Respiratory Diseases, China-Japan Friendship Hospital, Yinghuayuan East Street, Chao-yang District, Beijing, China; 4 Department of Pulmonary and Critical Care Medicine, China-Japan Friendship Hospital, Yinghuayuan East Street, Chao-yang District, Beijing, China; 5 Department of Respiratory Medicine, Yan’an Hospital Affiliated to Kunming Medical University, Renmin East Road, Kunming City, Yunnan Province China; 6 Department of Infectious Disease, Beijing Jishuitan Hospital, Xinjiekou East Street, Xi-cheng District, Beijing, China; 7 Department of Respiratory Medicine, Beijing Huimin Hospital, Youanmen Street, Xi-cheng District, Beijing, China; 8 Department of Respiratory Medicine, Linzi District People’s Hospital, Huangong Road, Zibo City, Shandong Province China; 9 Department of Respiratory Medicine, Beijing Luhe Hospital, Capital Medical University, Xinhua South Road, Tongzhou District, Beijing, China; 10 Department of Pulmonary and Critical Care Medicine, Weifang No. 2 People’s Hospital, Yuanxiao Street, Weifang City, Shandong Province China; 11 Department of Respiratory Medicine, Shandong University Affiliated Qilu Hospital (Qingdao), Hefei Road, Qingdao City, Shandong Province China; 12 Department of Respiratory Medicine, The 2nd Hospital of Beijing Corps, Chinese Armed Police Forces, Yuetan North Street, Xi-cheng District, Beijing, China; 13 Department of Infectious Disease, Qingdao University Medical College Affiliated Yantaiyuhuangding Hospital, Yudong Road, Yantai City, Shandong Province China; 14 Department of Respiratory Medicine, Rizhao Chinese Medical Hospital Affiliated to Shandong Chinese Medical University, Wanghai Road, Rizhao City, Shandong Province China; 15 Department of Respiratory Medicine, Beijing Hospital of Traditional Chinese Medicine Affiliated to Capital Medical University, Meishuguan Street, Dong-cheng District, Beijing, China; 16 Department of Occupational Medicine and Toxicology, Beijing Chao-Yang Hospital, Capital Medical University, Gongti South Road, Chao-yang District, Beijing, China; 17 Senior Director, Anti-infectives, Pfizer Inc, 500 Arcola Rd, F3203, Collegeville, PA 19426 USA; 18 Department of Pulmonary Medicine, Capital Medical University, Yinghuayuan East Street, Chao-yang District, Beijing, China

**Keywords:** Community-acquired pneumonia, Overtreatment, Adherence to guidelines, Elderly

## Abstract

**Background:**

Limited information exists on the clinical characteristics predictive of mortality in patients aged ≥65 years in many countries. The impact of adherence to current antimicrobial guidelines on the mortality of hospitalized elderly patients with community-acquired pneumonia (CAP) has never been assessed.

**Methods:**

A total of 3131 patients aged ≥65 years were enrolled from a multi-center, retrospective, observational study initiated by the CAP-China network. Risk factors for death were screened with multivariable logistic regression analysis, with emphasis on the evaluation of age, comorbidities and antimicrobial treatment regimen with regard to the current Chinese CAP guidelines.

**Results:**

The mean age of the study population was 77.4 ± 7.4 years. Overall in-hospital and 60-day mortality were 5.7% and 7.6%, respectively; these rates were three-fold higher in those aged ≥85 years than in the 65–74 group (11.9% versus 3.2% for in-hospital mortality and 14.1% versus 4.7% for 60-day mortality, respectively). The mortality was significantly higher among patients with comorbidities compared with those who were otherwise healthy. According to the 2016 Chinese CAP guidelines, 62.1% of patients (1907/3073) received non-adherent treatment. For general-ward patients without risk factors for Pseudomonas aeruginosa (PA) infection (*n* = 2258), 52.3% (1094/2090) were over-treated, characterized by monotherapy with an anti-pseudomonal β-lactam or combination with fluoroquinolone + β-lactam; while 71.4% of intensive care unit (ICU) patients (120/168) were undertreated, without coverage of atypical bacteria. Among patients with risk factors for PA infection (*n* = 815), 22.9% (165/722) of those in the general ward and 74.2% of those in the ICU (69/93) were undertreated, using regimens without anti-pseudomonal activity. The independent predictors of 60-day mortality were age, long-term bedridden status, congestive heart failure, CURB-65, glucose, heart rate, arterial oxygen saturation (SaO_2_) and albumin levels.

**Conclusions:**

Overtreatment in general-ward patients and undertreatment in ICU patients were critical problems. Compliance with Chinese guidelines will require fundamental changes in standard-of-care treatment patterns. The data included herein may facilitate early identification of patients at increased risk of mortality.

**Trial registration:**

The study was registered at ClinicalTrials.gov (NCT02489578).

**Electronic supplementary material:**

The online version of this article (10.1186/s12879-018-3098-5) contains supplementary material, which is available to authorized users.

## Background

Despite significant reductions in deaths due to community-acquired pneumonia (CAP) between 2009 and 2013, lower respiratory tract infections (LRTI) remain a leading cause of death in China, responsible for more than 19 deaths per 100,000 people per year [1]. In 2013, LRTI ranked 2nd among causes of global years of life lost [2]. CAP remains a significant threat, especially to the elderly, and it is the most common cause of infectious disease-related hospitalization and death [3, 4].

Among elderly patients with CAP, predictors of death include increased age [5–9], underlying comorbidity [5–7] and severity of illness [3, 6, 9]. Studies have reported mortality rates of elderly patients with CAP ranging from 6.4% to 33% [9–13], roughly doubling as age increased from 65 to 69 years to > 90 years [5]. An increasing number of studies suggest that adherence to CAP guidelines improves patient outcomes and decreases healthcare costs [14–20]. The analysis conducted by the Community-Acquired Pneumonia Organization (CAPO) International Study database showed that for hospitalized CAP patients aged ≥65 years, adherence to the 2007 Infectious Diseases Society of America (IDSA)/American Thoracic Society (ATS) guidelines was associated with a dramatically significant decrease in the time to achieve clinical stability, shorter length of stay (LOS) and lower overall mortality compared with non-adherent therapy [17]. Moreover, adherence was a cost-effective strategy for patients in general wards [20]. For patients hospitalized in the ICU, a retrospective study found that guideline-concordant therapy was associated with decreased mortality [21]. In contrast, studies by Rossio [22] and Wilson [23] found that adherence to guidelines did not affect mortality among patients with CAP.

As the life span of the global population continues to increase, the number of elderly patients hospitalized with CAP will increase accordingly, placing greater demands on available healthcare resources and highlighting the importance of understanding the epidemiology of CAP among the elderly. Existing studies about hospitalized CAP patients aged ≥65 years have largely focused on North American and European populations. Chinese adult CAP guidelines were most recently updated in April 2016; however, in clinical practice, to what degree physicians need to tailor their treatment approach to comply with such guidelines and whether these changes actually improve patient outcomes both require assessment. To address these important questions, a multicenter, retrospective observational study was conducted by the CAP-China network. The objectives of the study included a comparison of the common treatment patterns in the 2014 to 2016 guidelines and an investigation of the influences of age, comorbidity and antimicrobial regimens adherent to the current 2016 CAP guideline on the mortality of hospitalized elderly patients with CAP.

## Methods

### Study setting, design and participants

The current study was an observational study initiated by the CAP-China network. Data from CAP patients (International Classification of Diseases, tenth revision [ICD-10] codes (in the Additional file 1: Table S1)) admitted between January 1st, 2014 and December 31st, 2014 from 13 centers in seven cities of three provinces were collected with standard case report forms by a member of the investigation team trained to ensure consistency across the study (in Additional file 1: Table S2). Validation of data quality was performed by a second group of specially assigned researchers before the case was entered into the CAP-China database. Only those patients meeting all pre-defined inclusion/exclusion criteria were included in the analysis.

Inclusion/ Exclusion criteria and definitions.

Inclusion criteria included the following: (1) age ≥ 65 years; (2) one of the top five discharge diagnoses defined as CAP.

Exclusion criteria included the following: (1) hospital-acquired pneumonia (HAP); (2) active tuberculosis; (3) non-infectious diseases, such as pulmonary infarction, tumor or pulmonary edema; (4) acquired immune deficiency syndrome; (5) re-admission within 72 h after discharge.

CAP was defined as follows: (1) community onset; (2) presence of new infiltrate on chest X-ray or computed tomography scan together with at least one of the following: (i) new or increased cough (productive, non-productive or with a change in sputum characteristics) with or without dyspnea, chest pain or hemoptysis, (ii) fever, (iii) rales and/or signs of consolidation, (iv) peripheral WBC counts > 10,000 cells·mm^− 3^ or < 4000 cells·mm^− 3^, with or without a left shift toward immature forms.

Immunocompromised patients and those with healthcare-associated pneumonia (HCAP) were not excluded from the study to ensure that the data reflected the characteristics of a general population. Immunocompromised conditions were defined as: (1) solid-organ transplant; (2) stem cell transplant or bone marrow transplantation within one year of admission, or at any time following transplantation for those with graft versus host disease; (3) chemotherapy for hematological disease or solid-tumor malignancy within six months of admission [24] or neutropenia < 500 cells·m^− 3^; (4) chest radiation therapy within one month of admission [25]; (5) autoimmune disease receiving immunosuppressive therapy within three months of admission [i.e., oral prednisone ≥10 mg·d^− 1^ for more than 3 weeks or the equivalent; cyclosporine or azathioprine use, and methotrexate use larger than 12.5 mg·week^− 1^ [26]; biological modifiers such as etanercept and infliximab]; or (6) splenectomy.

HCAP was defined as (1) hospitalization for two or more days in the 90 days before admission; (2) outpatient infusion therapy or chemotherapy or home wound care in the previous 30 days; (3) admission from a nursing home or long-term care facility; or (4) chronic dialysis in a hospital or clinic [27].

### Data collection

The following parameters were collected: age, sex, date of admission/discharge/death, preexisting comorbidities, long-term bedridden status, history of aspiration, clinical signs on admission (body temperature, respiratory rate, heart rate and blood pressure), confusion, SaO_2_, laboratory values [white blood cell (WBC) counts, blood albumin, blood creatinine, blood urea nitrogen (BUN), sodium and blood glucose], microbiological information (Additional file 1: S3), chest radiography (number of lobes affected and pleural effusion), septic shock, ICU admission, mechanical ventilation (MV), LOS and antimicrobial therapy of pre-hospitalization and after admission.

Severity of illness within 24 h after admission was determined using the pneumonia severity index (PSI) [28] and the CURB-65 scoring system [29]. Patients were stratified into low-risk, moderate-risk and high-risk groups using the following criteria: PSI score: low risk = classes I-III, moderate risk = class IV and high risk = class V; CURB-65: low risk = class 1, moderate risk = class 2 and high risk = classes 3–5.

Consistency with Chinese CAP guidelines (2016 version).

The initial antimicrobial drugs administered within the first 48 h were evaluated and categorized as adherence, overtreatment or undertreatment with regard to the 2016 Chinese CAP guideline [30]. Empirical antimicrobial therapy consistent with recommendations in the guideline was defined as adherence (Additional file 1: Table S4). Regimens covering less than what is recommended in the guideline were defined as undertreatment, while regimens in excess of the recommendations were considered overtreatment.

For instance, patients without risk factors for Pseudomonas aeruginosa (PA) infection in the general wards who received anti-pseudomonal β-lactam were considered to be overtreated and who received penicillin or first/second generation cephalosporins were considered to be undertreated. Similarly, patients without risk factors for PA in the ICU treated with a respiratory fluoroquinolone alone were considered undertreated; while combination with anti-pseudomonal β-lactam were considered to be overtreated. Patients with risk factors for PA who received monotherapy or combination without anti-pseudomonal activity were considered to be undertreated.

### Statistical analyses

Data are presented as frequencies or percentages for categorical variables and the mean ± standard deviation for continuous variables. CAP patients were divided into three age groups (65–74 years, 75–84 years and ≥ 85 years); the characteristics of each group were compared using the Χ^2^ test for categorical variables and ANOVA tests for continuous variables.

In-hospital mortality and risk factors for 60-day mortality were evaluated. Variables showing significant difference in univariate analysis (*p* < 0.20) were included in multivariate logistic analysis, and a stepwise forward model was used to select independent risk factors. The 95% confidence intervals (CIs) and level of significance were reported. Multivariate Cox regression models were used to compare the difference in 60-day mortality between different groups, with all the other factors impacting the outcome being adjusted, except for the group factor. Cumulative survival curves for 60-day mortality were described, stratified by treatment, age group and number of comorbidities.

All data were analyzed with SPSS (version 20, IBM Corp., New York, USA); *p* < 0.05 was considered statistically significant.

## Results

### Study population and clinical characteristics

Of the 6056 CAP patients fulfilling the inclusion and exclusion criteria, more than 50% of the patients (*n* = 3131) were 65 years or older and underwent further analysis. The screening was cited from another study by our team [31]. Baseline characteristics of the patient cohorts grouped by age are provided in Table 1. The mean age of the study population was 77.4 ± 7.4 years; males accounted for slightly more than half (54.5%) of the patients. This study found that 90.6% of patients had at least one underlying disease; of these, two-thirds had two or more identified comorbidities. The most common underlying diseases were cardiovascular disease, chronic pulmonary disease and cerebral vascular disease.

**Table 1:** Characteristics of hospitalized CAP patients over 65 years by age group (*n* = 3131)

Characteristics	65–74 y (*n* = 1134)	75–84 y (*n* = 1449)	≥ 85 y (*n* = 548)
Age(years, *n* = 3131)	66.43 ± 2.87	79.43 ± 2.78	88.54 ± 3.38
Male sex(*n* = 3131)	633(55.8)	791(54.6)	281(51.3)
Aspiration(*n* = 3131)	72(6.3)	141(9.7)	110(20.1)
Long-term bedridden status(*n* = 3131)	57(5.0)	117(8.1)	92(16.8)
Underlying conditions(*n* = 3131)	998(88.0)	1318(91.0)	521(95.1)
Cardiovascular disease	615 (54.2)	935(64.5)	400(73.0)
Hypertension	488(43.0)	749(51.7)	302(55.1)
Ischemic heart disease	263(23.2)	493(34.0)	244(44.5)
Congestive heart failure	65(5.7)	101(7.0)	35(6.4)
Chronic respiratory disease	359(31.7)	391(27.0)	125(22.8)
COPD	210(18.5)	275(19.0)	94(17.2)
Bronchiectasis	153(13.5)	112(7.7)	36(6.6)
Asthma	85(7.5)	29(4.4)	13(2.4)
Cerebral vascular disease	191(16.8)	412(28.4)	211(38.5)
Diabetes mellitus	224(19.8)	316(21.8)	111(20.3)
Malignancy	90(7.9)	122(8.4)	40(7.3)
Chronic renal disease	47(4.1)	76(5.2)	32(5.8)
Dementia	8(0.7)	24(1.7)	24(4.4)
Chronic liver disease	15(1.3)	20(1.4)	4(0.7)
History of hospitalization for CAP in previous year	80(7.1)	124(8.6)	59(10.8)
HCAP (*n* = 3131)	195(17.2)	231(15.9)	100(18.2)
Immunocompromise^a^ (*n* = 3131)	37(3.3)	27(1.9)	6(1.1)
Multilobe infiltration (*n* = 3131)	458(40.4)	676(46.7)	273(49.8)
Pleural effusion (*n* = 3131)	221 (19.5)	387(26.7)	176(32.1)
CURB-65 (*n* = 3025 ^b^)			
1	711(64.9)	769(55.6)	234(42.7)
2	322(29.4)	455(32.9)	198(36.1)
3–5	62(5.7)	158(11.4)	116(21.2)
PSI (*n* = 1714 ^c^)			
I-III	453(73.7)	395(49.8)	84(27.5)
IV	131(21.3)	320(40.4)	149(48.7)
V	31(5.0)	78(9.8)	73(23.9)
Need for MV (*n* = 3131)	87(7.7)	129(8.9)	57(10.4)
Non-invasive MV	62(5.5)	91(6.3)	40(7.3)
invasive MV	42(3.7)	52(3.6)	23(4.2)
ICU admission(*n* = 3131)	68(6.0)	126(8.7)	75(13.7)
Outcome			
Hospital LOS (days) (*n* = 3131)	12.73 ± 9.62	13.29 ± 9.61	15.21 ± 12.78
In-hospital mortality(*n* = 3131)	36(3.2)	78(5.4)	65(11.9)
60-day mortality(*n* = 3011^d^)	53(4.7)	97(6.7)	77(14.1)

*Abbreviations*: *COPD* chronic obstructive pulmonary disease, *HCAP* healthcare-associated pneumonia, *PSI* pneumonia severity index, *MV* mechanical ventilation, *ICU* intensive care unit, *LOS* length of stay

^a^defined as (1) solid-organ transplant recipients; (2) stem cell transplant recipients or bone marrow transplantation within one year of admission, or at any time following transplantation for those with graft versus host disease; (3) patients undergoing chemotherapy for hematological diseases or solid-tumor malignancies within six months of admission, or neutropenia < 500 cells·m^−3^; (4) chest radiation therapy within one month of admission; (5)autoimmune disease receiving immunosuppressive therapy within three months of admission (i.e., oral prednisonean  ≥ 10 mg•d^-1^ for more than 3 weeks or the equivalent); (6) splenectomy

^b^ Figure of urea nitrogen was missing in 106 cases

^c^ The total number of patients with a complete data of PSI score

^d^ Loss to follow-up for patients was 62 cases. Data on empirical antimicrobial regimens in 49 patients were missing. 3 patients in general ward administered antifungal agents and 6 patients in ICU administered antipseudomonal β-lactam plus antifungal agents were ruled out

Patients aged ≥85 years were more likely to have pleural effusion or multi-lobe infiltration and be admitted to ICU than those aged 65–84 years. However, there were no significant differences in the need for MV, either non-invasive or invasive.

The severity of pneumonia increased significantly with age. More than half of the patients had low-risk CURB-65 or PSI scores, and less than 11% of the patients were high-risk.

### Pathogens

Etiology was defined in 438 (14.0%) patients; PA was the most common pathogen in 20.1% of the isolates (99/492), with *Klebsiella pneumonia* in 15.2% (75/492), *Escherichia coli* in 9.8% (48/492), *Acinetobacter* in 8.3% (41/492), *Staphylococcus aureus* in 6.9% (34/492), *Streptococcus pneumonia* in 3.3% (16/492), viruses in 14.2% (70/492) and atypical pathogens in only 0.6% (3/492).

### Antimicrobial regimen

In this study, 42.3% (1323/3131) of the included patients had a documented history of pre-hospital medication; in 98 cases, the medication record was unknown. The route of administration was intravenous in 67.4% (826/1225) of patients, oral in 14.3% (175/1225) and unknown in 18.3% (224/1225). The most common medication regimen was monotherapy with a β-lactam (45.1%, 552/1225); of these, anti-pseudomonal β-lactam alone accounted for 26.4% (146/552). Monotherapy with fluoroquinolones (18.9%, 231/1225) as well as fluoroquinolones plus cephalosporins (18.0%, 220/1225) were other common pharmacotherapeutic choices. Among patients in the current study, the total use of anti-pseudomonal β-lactam was 19.1% (234/1225) and fluoroquinolones 39.9% (489/1225).

Of the 3131 cases studied, data on the empirical use of antimicrobial drugs were missing in 49 cases, and 9 cases were ruled out due to the administration of antifungal agents. Ultimately, 3073 cases were included in the final analysis.

2258 patients (2090 in general ward and 168 in ICU) had no risk factors for PA infection such as bronchiectasis, HCAP or immunocompromised conditions; among them 35.3% (1462/2258) received antimicrobial treatment that was consistent with the Chinese CAP guideline (2016 version). Monotherapy with a β-lactam or fluoroquinolone was the most common regimen. Overtreatment was much more common among general-ward patients (52%, 1094/2090) than ICU patients (28.0%, 47/168) (Table 2).

**Table 2:** Application of 2016 Chinese CAP guideline in hospitalized patients over 65 years without risk factors of *Pseudomonas aeruginosa* infection (*n* = 2258)

Regimen	General ward inpatients (*n* = 2090)	ICU patients (*n* = 168)
Consistent with guideline	795(38.0)	1(0.6)
β-lactam	415(19.8)	0(0)
Fluoroquinolone	312(14.9)	0(0)
β-lactam + macrolide	68(3.3)	0(0)
β-lactam + fluoroquinolone	0(0)	1(0.6)
Undertreated by guideline	201(9.6)	120(71.4)
β-lactam	153(7.3) ^a^	92(54.8)^b^
β-lactam + macrolide	21(1.0)^a^	1(0.6)
Macrolide	15(0.7)	0(0)
Fluoroquinolone +/− β-lactam^c^	0(0)	26(15.5)
Other combination	12(0.6)	1(0.6)
Overtreated by guideline	1094(52.3)	47(28.0)
Antipseudomonal β-lactam^d^	530(25.4)^#^	0(0)
Fluoroquinolone +β-lactam/ other^e^	451(21.6) ^##^	37(22.0)^*^
Antipseudomonal β-lactam^d^ + macrolide	46(2.2) ^###^	5(3.0)^**^
Antipseudomonal β-lactam^d^ + other^e^	39(1.9) ^####^	2(1.2) ^***^
Fluoroquinolone + macrolide	12(0.6)	0(0)
β-lactam +quinolone+ other^e^	8(0.3)	3(1.8) ^****^
β-lactam +macrolide+ fluoroquinolone /other^e^	8(0.3)	0(0)

Data on empirical antimicrobial regimens in 37 patients were missing. Three patients in the general ward who were administered antifungal agents, and 6 patients in the ICU who were administered antipseudomonal β-lactam plus antifungal agents were ruled out

β-lactam^a^ = penicillin, first / second generation cephalosporins

β-lactam^b^ = second/ third generation cephalosporins (*n* = 5), cephamycins (*n* = 12) and antipseudomonal β-lactam (*n* = 75, including carbapenem 25)

β-lactam^c^ = second generation cephalosporins (*n* = 6) and cephamycins (*n* = 7)

Antipseudomonal β-lactam^d^ = piperacillin/tazobactam, ticarcillin/clavulanic acid, mezlocillin/sulbactam, cefoperazone/sulbactam, ceftazidime, cefoperazone, cefepime, carbapenem (imipenem / cilastatin and meropenem)

other^e^ = imidazoles, tetracyclines, aminoglycoside, lincomycin, fosfomycin and glycopeptides

^#^ carbapenem = 108

^##^ Antipseudomonal β-lactam = 260 (carbapenem = 36), other β-lactam = 182, other^e^ = 9

^###^ carbapenem = 11

^####^ carbapenem+glycopeptide =6, carbapenem+other =6, other antipseudomonal β-lactam+other =27

* Antipseudomonal β-lactam = 36 (carbapenem = 10); ** carbapenem = 2

*** carbapenem+glycopeptide = 2; **** carbapenem = 2

For patients with risk factors for PA infection (*n* = 815), 45.4% (*n* = 370) received adherent treatment. The most common regimen was an anti-pseudomonal β-lactam alone for the general-ward patients, while anti-pseudomonal β-lactam plus fluoroquinolone was the most common regimen for ICU patients (Table 3).

**Table 3:** Application of 2016 Chinese CAP guidelines in hospitalized patients over 65 years with risk factors of *Pseudomonas aeruginosa* infection (*n* = 815)

Regimen	General ward inpatients (*n* = 722)	ICU patients (*n* = 93)
Consistent with guideline	348(48.2)	22(23.7)
Antipseudomonal β-lactam	247(34.2)	0(0)
Antipseudomonal fluoroquinolone	85(11.8)	0(0)
Antipseudomonal β-lactam + macrolide	16(2.2)	0(0)
Antipseudomonal β-lactam +fluoroquinolone	0(0)	21(22.6)
Antipseudomonal β-lactam+ aminoglycosides	0(0)	1(1.1)
Undertreated by guideline	165(22.9)	69(74.2)
β-lactam	132(18.3)	10(10.8)
Antipseudomonal β-lactam	0(0)	32(34.4)
Macrolide	5(0.7)	0(0)
β-lactam + macrolide	23(3.2)	1(1.1)
Antipseudomonal fluoroquinolone +/− β-lactam/ other	0(0)	9(9.7)
Antipseudomonal β-lactam + macrolide	0(0)	4(4.3)
Antipseudomonal fluoroquinolone + macrolide	0(0)	2(2.2)
Other combination	5(0.7)	11(11.8)
Overtreated by guideline	209(28.9)	2(2.2)
Antipseudomonal β-lactam + antipseudomonal fluoroquinolone + macrolide /other	6(0.8)	2(2.2)
Antipseudomonal β-lactam + antipseudomonal fluoroquinolone	96(13.3)	0(0)
Antipseudomonal fluoroquinolone +β-lactam	65(9.0)	0(0)
Antipseudomonal fluoroquinolone+ macrolide	5(0.7)	0(0)
Antipseudomonal fluoroquinolone + other	13(1.8)	0(0)
Antipseudomonal β-lactam + other	24(3.3)	0(0)

Data on empirical antimicrobial regimens in 12 patients were missing

other = imidazoles, lincomycin, fosfomycin, glycopeptides and antifungal agents

### Clinical outcomes and impacts of age, comorbidities and antimicrobial regimen

The overall in-hospital mortality of CAP patients aged ≥65 years was 5.7% (179/3131) with no significant difference between men (6.2%) and women (5.1%). Among them, 8.6% patients (269/3131) were admitted to the ICU, and 25.3% (68/269) died during hospitalization; a total of 75 patients (27.9%) died within 60 days of admission.

Mortality increased along with disease severity scores calculated based on parameters on admission. Using the CURB-65 to classify disease severity, in-hospital mortality and 60-day mortality were 2.1% and 3.3% for Class 1 patients, 7.0% and 8.4% for Class 2 patients, and 21.2% and 24.7% for Class 3–5 patients, respectively. Similarly, mortality increased directly from low-risk to high-risk class according to PSI score (*n* = 1714).

**Fig. 1:**
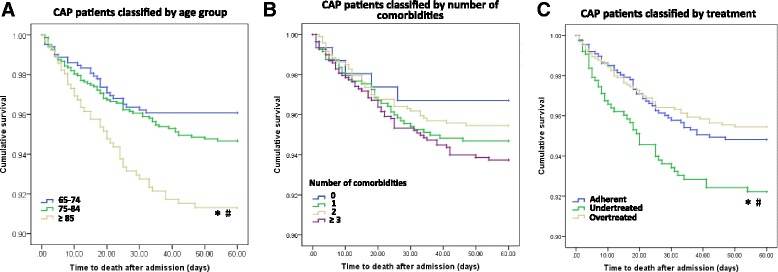
Cumulative survival curves classified by age group, number of comorbidity and treatment in hospitalized elderly community-acquired pneumonia patients (*n* = 3011). **a** classification by age group, compared with the 65–74-year age group (*n* = 1085), *p = 0.002; compared with the 75–84-year age group (*n* = 1401), ^#^*p* = 0.004. **b** classification by number of comorbidities. Cases with the numbers of comorbidities were 287, 913, 871 and 940. **c** classification by treatment compared with patients with adherent treatment (*n* = 1147), ^*^p = 0.012; compared with patients with overtreatment (*n* = 1330), ^#^*p* = 0.057

In addition to disease severity on admission, age is another important factor for death. In-hospital mortality and 60-day mortality in those aged ≥85 years were more than three times higher than in those aged 65–74 years. The cumulative survival curve further showed that 60-day mortality significantly increased with age (Fig. 1a), but the mortality of elderly patients with CAP was not associated with the number of comorbidities (Fig. 1b).

Further, we assessed the effect of adherence to the Chinese CAP guideline of antibiotic therapy on mortality and found that undertreatment was associated with an increased mortality rate. However, overtreatment with broad-spectrum antimicrobial regimen did not lower the mortality (Fig. 1c). These results suggest that physicians may need to adjust their treatment strategies to adhere to current guidelines.

### Predictive factors of 60-day mortality

Logistic regression analysis allowed us to further explore the factors predictive of mortality in elderly patients with CAP. All the potential factors screened in the univariate analysis with *p* < 0.20 variables were included in the regression model (Additional file 1: Table S5). Long-term bedridden status, congestive heart failure, CURB-65, glucose, heart rate and age were independent poor prognostic factors of 60-day mortality, while increased SaO_2_ and albumin levels were associated with better prognosis (Table 4). For further sensitivity analysis, we listed all of the factors in supplementary Table 4 that could influence patients’ outcome in the multivariable logistic regression analysis; similar results were obtained and are listed in supplementary S6.

**Table 4:** Multivariable analysis of predictive factors of 60-day mortality in elderly CAP patients (*n* = 3, 011)

Predictive factors	OR	95%CI	*p* value
Long-term bedridden status	2.1	1.2–3.8	0.009
Congestive heart failure	2.1	1.1–4.11	0.031
CURB-65	1.9	1.5–2.4	< 0.001
Glucose	1.06	1.01–1.12	0.037
Heart rate	1.04	1.01–1.07	0.010
Age	1.01	1.01–1.03	0.013
SaO_2_	0.98	0.96–0.99	0.023
Albumin	0.94	0.90–0.97	< 0.001

*Abbreviations*: *WBC* white blood cell, *SaO*_*2*_ arterial oxygen saturation

Loss to follow-up for patients was 62 cases. Data on empirical antimicrobial regimens in 49 patients were missing. Three patients in the general ward who were administered antifungal agents and 6 patients in the ICU who were administered antipseudomonal β-lactam plus antifungal agents were ruled out

## Discussion

This is the first retrospective multicenter study to assess the influences of age, comorbidity and antimicrobial treatment on outcomes of hospitalized elderly patients with CAP in China. The major findings of our study were as follows: 1) the overall in-hospital mortality and 60-day mortality rates were relatively low, 5.7% and 7. 6%, respectively, and age was the independent prognostic factor most associated with mortality; 2) 60% of patients had two or more comorbidities; congestive heart failure and long-term bedridden condition were independent risk factors of 60-day mortality; 3) 62.1% of patients received non-adherent treatment with antibiotics active for this genus.

In the present study, the breakdown of age was similar to data reported from a study in Spain, which enrolled 2149 patients aged ≥65 years [32]. The comorbidity ratios in our study were higher than those previously reported [5, 32, 33].

The proportion of non-adherent regimens (62.1%) in our study was significantly greater than in the CAPO study [17] and other studies [20–22]. For patients without risk factors for PA infection in general wards, overtreatment with anti-pseudomonal β-lactam regimens approached 80%, far greater than the data reported by Egger, which showed only 1% [20]. The wide-spread use of *Pseudomonas*-active carbapenem (other than ertapenem) was critical. This inappropriate use of carbapenems placed unnecessary pressure on bacteria to develop resistance, thus increasing the risk of Carbapenem-resistant *Enterobacteriaceae* (CRE). The reason for widespread overtreatment of general ward patients is unclear but is likely multifactorial. Clearly, part of the reason is that patients included in this study were treated before Chinese CAP guidelines were published. For physicians, choice of treatment should follow guidelines, and therefore, significant changes in treatment patterns are required. Furthermore, some patients may have received prior outpatient antibiotics that were consistent with the guidelines but that failed. A patient’s intent to be cured as soon as possible after admission and the burden of an average day of hospitalization may force the physician to treat more aggressively on admission, making the “first-choice, empiric therapy” more consistent with a second-line regimen. We can also see a low detection rate (14.0%) of pathogens and high detection rate of gram-negative bacilli (71.3%) in our data; pre-hospitalization antibiotic abuse and real or perceived risk of multi-drug-resistant pathogens may also have prompted physicians to overtreat to avoid failure. Better regional epidemiology data are critical to avoid the over-use of broad-spectrum agents.

Our data did not identify a relationship between non-compliance (either overtreatment or undertreatment) and mortality, as in previously published studies [22, 23]. There are a number of explanations for this observation. First, the best predictor of mortality was the severity of illness. In our study, CURB-65 was also confirmed as a risk factor of mortality. Thus, if guidelines did not capture patient severity to a sufficient degree, antibiotic choices might be appropriate for the patient but still be non-compliant with guidelines. Furthermore, patients transferred from other facilities or those who received outpatient antibiotics had an “initial” regimen influenced substantially by prior choices that were unsuccessful. Second, 2016 CAP guidelines might have been inferior to the 2014 standard-of-care. This seems unlikely but cannot be ruled out based on these data. Finally, this study gathered data only on the reported initial antimicrobial therapy, which may have been subsequently changed.

The overall mortality was lower than prior studies from the United States [5, 6, 11, 13], Europe [12] and Japan [9], due to a higher proportion of patients with lower risk scores. Meanwhile, compared with Malaysia, Indonesia and the Philippines [34], the mortality was higher. In fact, approximately 90% of hospitalized elderly CAP patients in China were of low to moderate risk. In a population-based study including 590 patients over 65 years old, the in-hospital mortality of low to moderate risk was 12.0% classified by CURB-65 score [35]. Luna and coworkers [6] reported an overall 30-day mortality rate of 8.2% in CAP patients, although they found little increase with age until patients were aged over 80 years, in contrast to our data. However, it is also possible that we were unable to fully assess the patients’ severity based on this chart review, and patients did receive appropriate site-specific care and initial antibiotic treatment on admission, especially based on possible prior antibiotic treatment [36, 37], resulting in appropriate infection control with reduced mortality rates.

We found many factors associated with a higher mortality rate; however, in the multivariate analysis, only 8 factors remained associated with increased 60-day mortality. Age was one of them, similar to the findings of previous studies [5, 13, 32, 33, 38]. It is widely acknowledged that the PSI is greatly affected by age. Given that the mean age of patients in this study was 77.4 ± 7.4 years; this would significantly affect the severity ratings for patients older than 75 years. Congestive heart failure was the only predictor of comorbidities related with mortality. Riquelme [13] reported that bedridden status was associated with a worse prognosis among elderly patients with CAP, consistent with our finding. CURB-65 is the most commonly used scoring system to assess severity of pneumonia. Data from Ochoa-Gondar O [35] demonstrated that CURB-65 was a good rule in predicting short-term mortality among elderly CAP patients. Data in our study showed that increased CURB-65 score was associated with increased 60-day mortality. Glucose level and heart rate were also risk factors of mortality. Higher glucose levels suggest that infection is more difficult to control. Heart rate is a component of PSI or SMART-COP score [39]; usually, the faster the heart rate, the more severe the infection. Although serum albumin was an acute phase reactant, the production of which dropped sharply by the liver in an inflammatory environment, it was not a good marker of nutrition status, and our study found that albumin was a protective factor against mortality, coinciding with figures from Hong Kong [40]. SaO_2_ was another protective factor against mortality; low oxygen clearly suggests more severe infections.

Some limitations of the present study deserve consideration. First, this was a retrospective observational study. Inevitably, we encountered some missing data, making it impossible to calculate PSI scores in nearly half of all patients. While a potential confounder, CURB-65 scores were less affected by missing data, but our study had a slightly higher proportion of patients with less severe pneumonia (CURB score < 2) compared with previous studies [29, 41]. Second, some cases of pneumonia may have been overlooked due to miscoding or because requiring a primary pneumonia ICD-10 code was too restrictive. Third, antimicrobial therapy was initiated prior to hospitalization for many patients; therefore, the initial antimicrobial regimen captured in this study was not necessarily the actual initial antimicrobial regimen that the patient received. This is especially true for patients transferred from other hospitals who were not excluded from the study.

## Conclusions

This study is the first attempt to describe the clinical characteristics and burden of hospitalizations for CAP among elderly patients in China. Predictive factors identified in the present study may help physicians recognize CAP patients at increased risk of mortality in the future. Most importantly, we noted that standard-of-care treatment regimens in 2014 were substantially different from current 2016 Chinese CAP guidelines, which suggests an urgent need for education. Although we found no relationship between antibiotic treatment and mortality, further prospective data should be gathered before conclusions can be drawn. Finally, the apparent overreliance on carbapenem antibiotics creates unfavorable pressure on bacteria, especially *Enterobacteriaceae*, to become resistant to this important class of antibiotics. Given the global increase in carbapenem-resistant Gram-negative pathogens, this is a potential nightmare scenario should these pathogens become established in Chinese populations and within the healthcare system.

## Additional file

Additional file 1: **Table S1** International Classification of pneumonia, tenth revision [ICD-10] codes. **Table S2** Details of participating centers. **S3** Definition of microbiological criteria of CAP. **Table S4** Recommendation of empirical therapy according to the 2016 Chinese CAP guideline. **Table S5** Univariate analysis of prognostic factors of 60-day mortality in elderly CAP patients (*n* = 3011). **Table S6** Multivariable analysis of predictive factors of 60-day mortality in elderly CAP patients (*n* = 3011). (DOCX 44 kb)

## Abbreviations

BUN: blood urea nitrogen
CAP: community-acquired pneumonia
CAPO: Community-Acquired Pneumonia Organization
CIs: confidence intervals
CRE: Carbapenem-resistant *Enterobacteriaceae*
HAP: hospital acquired pneumonia
HCAP: healthcare-associated pneumonia
ICD-10: International Classification of Diseases, tenth revision
ICU: intensive care unit
IDSA/ ATS: Infectious Diseases Society of America /American Thoracic Society
LOS: length of stay
LRTI: lower respiratory tract infections
MV: mechanical ventilation
PA: *Pseudomonas aeruginosa*
PSI: pneumonia severity index
SaO_2_: arterial oxygen saturation
SMART-COP: Systolic blood pressure, Multilobar infiltrate, Albumin, Respiratory Rate, Tachycardia, Confusion, low Oxygen, low PH
WBC: white blood cell

## References

[1] Zhou M, Wang H, Zhu J, Chen W, Wang L, Liu S (2016). Cause-specific mortality for 240 causes in China during 1990-2013: asystematic subnational analysis for the global burden of disease Study2013. Lancet.

[2] GBD 2013 Mortality and Causes of Death Collaborators (2015). Global, regional, and national age-sex specific all-cause and cause-specific mortality for 240 causes of death, 1990-2013: a systematic analysis for the global burden of disease study 2013. Lancet.

[3] Welte T, Torres A, Nathwani D (2012). Clinical and economic burden of community-acquired pneumonia among adults in Europe. Thorax.

[4] Jain S, Self WH, Wunderink RG, Fakhran S, Balk R, Bramley AM (2015). Community-acquired pneumonia requiring hospitalization among U.S. adults. N Engl J Med.

[5] Kaplan V, Angus DC, Griffin MF, Clermont G, Scott Watson R, Linde-Zwirble WT (2002). Hospitalized community-acquired pneumonia in the elderly: age- and sex-related patterns of care and outcome in the United States. Am J Respir Crit Care Med.

[6] Luna CM, Palma I, Niederman MS, Membriani E, Giovini V, Wiemken TL (2016). The impact of age and comorbidities on the mortality of patients of different age groups admitted with community-acquired pneumonia. Ann Am Thorac Soc.

[7] Fernandez-Sabe N, Carratala J, Roson B, Dorca J, Verdaguer R, Manresa F (2003). Community- acquired pneumonia in very elderly patients: causative organisms, clinical characteristics and outcomes. Medicine.

[8] Eurich DT, Marrie TJ, Minhas-Sandhu JK, Majumdar SR (2015). Ten-year mortality after community-acquired pneumonia. A prospective cohort. Am J Respir Crit Care Med.

[9] Takayanagi N, Hara K, Tokunaga D (2006). Etiology and outcome of community-acquired pneumonia in relation to age and severity in hospitalized adult patients. Nihon Kokyuki Gakkai Zasshi.

[10] Klausen HH, Petersen J, Lindhardt T, Bandholm T, Hendriksen C, Kehlet H (2012). Outcomes in elderly Danish citizens admitted with community-acquired pneumonia. Regional differencties, in a public healthcare system. Respir Med.

[11] Venkatesan P, Gladman J, Macfarlane JT, Barer D, Berman P, Kinnear W (1990). A hospital study of community acquired pneumonia in the elderly. Thorax.

[12] Garcia-Ordonez MA, Garcia-Jimenez JM, Paez F, Alvarez F, Poyato B, Franquelo M (2001). Clinical aspects and prognostic factors in elderly patients hospitalised for community-acquired pneumonia. Eur J Clin Microbiol Infect Dis.

[13] Riquelme R, Torres A, El-Elbiary M, de la Bellacasa JP, Estruch R, Mensa J (1996). Community-acquired pneumonia in the elderly: a multivariate analysis of risk and prognostic factors. Am J Respir Crit Care Med.

[14] Dean NC, Bateman KA, Donnelly SM, Silver MP, Snow GL, Hale D (2006). Improved clinical outcomes with utilization of a community-acquired pneumonia guideline. Chest.

[15] Capelastegui A, Espana PP, Quintana JM, Gorordo I, Ortega M, Idoiaga I (2004). Improvement of process-of-care and outcomes after implementing a guideline for the management of community-acquired pneumonia: a controlled before-and-after design study. Clin Infect Dis.

[16] Nathwani D, Rubinstein E, Barlow G, Davey P (2001). Do guidelines for community-acquired pneumonia improve the cost-effectiveness of hospital care?. Clin Infect Dis.

[17] Arnold FW, LaJoie AS, Brock GN, Peyrani P, Rello J, Menéndez R (2009). Improving outcomes in elderly patients with community-acquired pneumonia by adhering to national guidelines: community-acquired pneumonia organization international cohort study results. Arch Intern Med.

[18] Frei CR, Restrepo MI, Mortensen EM, Burgess DS (2006). Impact of guideline-concordant empiric antibiotic therapy in community-acquired pneumonia. Am J Med.

[19] Mortensen EM, Restrepo M, Anzueto A, Pugh J (2004). Effects of guideline-concordant antimicrobial therapy on mortality among patients with community-acquired pneumonia. Am J Med.

[20] Egger ME, Myers JA, Arnold FW, Pass LA, Ramirez JA, Brock GN (2016). Cost effectiveness of adherence to IDSA/ATS guidelines in elderly patients hospitalized for Community-Aquired Pneumonia. BMC Med Inform DecisMak.

[21] Frei CR, Attridge RT, Mortensen EM, Restrepo MI, Yu Y, Oramasionwu CU (2010). Guideline-concordant antibiotic use and survival among patients with community-acquired pneumonia admitted to the intensive care unit. Clin Ther.

[22] Rossio R, Franchi C, Ardoino I, Djade CD, Tettamanti M, Pasina L (2015). Adherence to antibiotic treatment guidelines and outcomes in the hospitalized elderly with different types of pneumonia. Eur J Intern Med.

[23] Wilson BZ, Anzueto A, Restrepo MI, Pugh MJ, Mortensen EM (2012). Comparison of two guideline-concordant antimicrobial combinations in elderly patients hospitalized with severe community-acquired pneumonia. Crit Care Med.

[24] Mackall CL, Fleisher TA, Brown MR, Andrich MP, Chen CC, Feuerstein IM (1995). Age, thymopoiesis, and CD^4+^ T-lymphocyte regeneration after intensive chemotherapy. N Engl J Med.

[25] Uh S, Lee SM, Kim HT, Chung Y, Kim YH, Park C (1994). The effect of radiation therapy on immune function in patients with squamous cell lung carcinoma. Chest.

[26] Kanik KS, Cash JM (1997). Does methotrexate increase the risk of infection or malignancy?. Rheum Dis Clin N Am.

[27] American Thoracic Society (2005). Infectious diseases society of America. Guidelines for the management of adults with hospital acquired, ventilator-associated, and healthcare-associated pneumonia. Am J Respir Crit Care Med.

[28] Fine MJ, Auble TE, Yealy DM, Hanusa BH, Weissfeld LA, Singer DE (1997). A prediction rule to identify low-risk patients with community acquired pneumonia. N Engl J Med.

[29] Lim WS, van der Eerden MM, Laing R, Boersma WG, Karalus N, Town GI (2003). Defining community acquired pneumonia severity on presentation to hospital: an international derivation and validation study. Thorax.

[30] Cao B, Huang Y, She DY, Cheng QJ, Fan H, Tian XL, et al. Diagnosis and treatment of community-acquired pneumonia in adults: 2016 clinical practice guidelines by the Chinese thoracic society, Chinese Medical Association. Clin Respir J. 2017; 10.1111/crj.12674.

[31] Chen L, Zhou F, Li H, Xing X, Han X, Wang Y (2018). Disease characteristics and management of hospitalized adolescents and adults with Community-Acquired Pneumonia in China. BMJ Open.

[32] Cilloniz C, Polverino E, Ewig S, Aliberti S, Gabarrús A, Menéndez R (2013). Impact of age and comorbidity on cause and outcome in community-acquired pneumonia. Chest.

[33] Zalacain R, Torres A, Celis R, Blanquer J, Aspa J, Esteban L (2003). Community-acquired pneumonia in the elderly: Spanish multicentre study. Eur Respir J.

[34] Azmi S, Aljunid SM, Maimaiti N, Ali AA, Muhammad Nur A, De Rosas-Valera M (2016). Assessing the burden of pneumonia using administrative data from Malaysia, Indonesia, and the Philippines. Int J Infect Dis.

[35] Ochoa-Gondar O, Vila-Corcoles A, Rodriguez-Blanco T, Ramos F, de Diego C, Salsench E (2011). Comparison of three predictive rules for assessing severity in elderly patients with CAP. Int J Clin Pract.

[36] Torres A, Cillóniz C, Ferrer M, Gabarrús A, Polverino E, Villegas S (2015). Bacteraemia and antibiotic-resistant pathogens in community acquired pneumonia: risk and prognosis. Eur Respir J.

[37] Montull B, Menéndez R, Torres A, Reyes S, Méndez R, Zalacaín R (2016). Predictors of severe sepsis among patients hospitalized for community-acquired pneumonia. PLoS One.

[38] Jackson ML, Neuzil KM, Thompson WW, Shay DK, Yu O, Hanson CA (2004). The burden of community-acquired pneumonia in seniors: results of a population-based study. Clin Infect Dis.

[39] Charles PG, Wolfe R, Whitby M, Fine MJ, Fuller AJ, Stirling R (2008). SMART-COP: a tool for predicting the need for intensive respiratory or vasopressor support in community-acquired pneumonia. Clin Infect Dis.

[40] Ma HM, Tang WH, Woo J (2011). Predictors of in-hospital mortality of older patients admitted for community-acquired pneumonia. Age Ageing.

[41] Thiem U, Niklaus D, Sehlhoff B, Stückle C, Heppner HJ, Endres HG (2009). C-reactive protein, severity of pneumonia and mortality in elderly, hospitalised patients with community-acquired pneumonia. Age Ageing.

