# Supplementary material for: Effects of age, comorbidity and adherence to current antimicrobial guidelines on mortality in hospitalized elderly patients with community-acquired pneumonia
Source: BMC Infect Dis. 2018 Apr 24;18:192. doi: 10.1186/s12879-018-3098-5 (PMC5922029; doi:10.1186/s12879-018-3098-5)
Supplement: Supplementary file 1 — Table S1 International Classification of pneumonia, tenth revision [ICD-10] codes. Table S2 Details of participating centers. S3 Definition of microbiological criteria of CAP. Table S4 Recommendation of empirical therapy according to the 2016 Chinese CAP guideline. Table S5 Univariate analysis of prognostic factors of 60-day mortality in elderly CAP patients (n = 3011). Table S6 Multivariable analysis of predictive factors of 60-day mortality in elderly CAP patients (n = 3011). (DOCX 44 kb) [file 12879_2018_3098_MOESM1_ESM.docx]

**Table S1. International Classification of pneumonia, tenth revision [ICD-10] codes**

| Influenza with pneumonia, other influenza virus identified | J10.0 |
| --- | --- |
| Influenza with pneumonia, virus not identified | J11.0 |
| Virus pneumonia, not elsewhere classified | J12 |
| Adenoviral pneumonia | J12.0 |
| Respiratory syncytial virus pneumonia | J12.1 |
| Parainfluenza virus pneumonia | J12.2 |
| Other virus pneumonia | J12.8 |
| Viral pneumonia, unspecified | J12.9 |
| Pneumonia due to *Streptococcus pneumoniae* | J13 |
| Pneumonia due to *Haemophilus influenzae* | J14 |
| Bacterial pneumonia, not elsewhere classified | J15 |
| Pneumonia due to *Klebsiella pneumoniae* | J15.0 |
| Pneumonia due to *Pseudomonas spp.* | J15.1 |
| Pneumonia due to *Staphylococcus* | J15.2 |
| Pneumonia due to *Streptococcus spp*., group B | J15.3 |
| Pneumonia due to other *streptococci* | J15.4 |
| Pneumonia due to *Escherichia coli* | J15.5 |
| Pneumonia due to other aerobic Gram-negative bacteria | J15.6 |
| Pneumonia due to *Mycoplasma pneumoniae* | J15.7 |
| Other bacterial pneumonia | J15.8 |
| Bacterial pneumonia, unspecified | J15.9 |
| Pneumonia due to other infectious organisms, not elsewhere classified | J16 |
| Chlamydia pneumonia | J16.0 |
| Pneumonia due to other specified infectious organisms | J16.8 |
| Pneumonia in diseases classified elsewhere | J17* |
| Pneumonia in bacterial diseases classified elsewhere | J17.0* |
| Pneumonia in viral diseases classified elsewhere | J17.1* |
| Pneumonia in mycoses | J17.2* |
| Pneumonia in other diseases classified elsewhere | J17.8* |
| Pneumonia, organism unspecified | J18 |
| Bronchopneumonia, unspecified organism | J18.0 |
| Lobar pneumonia, unspecified | J18.1 |
| Hypostatic, pneumonia, unspecified | J18.2 |
| Other pneumonia, organism unspecified | J18.8 |
| Pneumonia, unspecified | J18.9 |
| Pulmonary mycobacterial infection | A31.0 |
| Pulmonary actinomycosis | A42.0 |
| Pulmonary nocardiosis | A43.0 |
| Legionnaires' disease | A48.1 |
| Varicella pneumonia | B01.2+ |
| Measles complicated by pneumonia | B05.1+ |
| Cytomegaloviral pneumonitis | B25.0+ |
| Pulmonary candidiasis | B37.1 |
| Acute pulmonary coccidioidomycosis | B38.0 |
| Acute pulmonary histoplasmosis capsulati | B39.0 |
| Acute pulmonary blastomycosis | B40.0 |
| Pulmonary paracoccidioidomycosis | B41.0 |
| Pulmonary sporotrichosis | B42.0+ |
| Invasive pulmonary aspergillosis | B44.0 |
| Other pulmonary aspergillosis | B44.1 |
| Pulmonary cryptococcosis | B45.0 |
| Pulmonary mucormycosis | B46.0 |

**Table S2: Details of participating centers**

| **Name of the hospital** | **Province, city** | **Rank of hospital** | **Teaching Hospital** | **Beds** | **Annual outpatient No.** | **Annual discharge No.** |
| --- | --- | --- | --- | --- | --- | --- |
| China-Japan Friendship Hospital | Beijing | 3^ry^ | Yes | 1610 | 2,689,745 | 59,308 |
| Beijing Jishuitan Hospital | Beijing | 3^ry^ | Yes | 1500 | 1,639,132 | 52,752 |
| Beijing Chao-Yang Hospital | Beijing | 3^ry^ | Yes | 1400 | 2,878,292 | 60,666 |
| **Beijing Luhe Hospital** | Beijing | 3^ry^ | Yes | 1042 | 4,207,092 | 36,371 |
| The 2^nd^ Hospital of Beijing Corps, Chinese Armed Police Forces | Beijing | 3^ry^ | Yes | 450 | 264,706 | 12,713 |
| **Beijing Huimin Hospital** | Beijing | 2^nd^ | Yes | 500 | 331,503 | 3,551 |
| Yantai Yuhuangding Hospital | Shangdong,  Yantai | 3^ry^ | Yes | 3000 | 1,930,717 | 101,360 |
| **Rizhao Chinese Medical Hospital** | Shangdong,  Rizhao | 3^ry^ | Yes | 1212 | 565,876 | 47,052 |
| **Qingdao Municipal Hospital** | ShanDong,  Qingdao | 3^ry^ | Yes | 1200 | 843,590 | 35,541 |
| **Shandong University Affiliated Qilu Hospital (Qingdao)** | ShanDong,  Qingdao | 3^ry^ | Yes | 1200 | 504,739 | 25,389 |
| **Linzi District People’s Hospital** | ShanDong,  Zibo | 3^ry^ | Yes | 1200 | 634,881 | 61,635 |
| **Weifang No.2 People’s Hospital** | Shangdong,  Weifang | 3^ry^ | Yes | 1006 | 168,200 | 19,790 |
| **Yan’an Hospital Affiliated to Kunming Medical University** | **Kunming,**  **Yan’an** | 3^ry^ | Yes | 1302 | 1,422,983 | 50,929 |

**Definition of rank of hospital in China:**

The 2^nd^ level hospital was defined as a hospital providing medical, prevention, health care and rehabilitation services to multiple communities (with a radius of population more than 100,000 peoples); the 3^ry^ level hospital was defined as a hospital providing medical service to the whole country beyond cities and provinces, with comprehensive medical, teaching and research ability.

**S3. Definition of microbiological criteria of CAP:**

**Definite**, if one of the following criteria was met:

1. Positive urinary antigen for *Legionella pneumophila* (LP, Binax Now L pneumophila urinary antigen test; Trinity Biotech, Bray, Ireland);
2. Positive urinary antigen for *Streptococcus pneumoniae* (Binax Now *S pneumoniae* urinary antigen test; Emergo Europe, The Netherlands);
3. Positive bacterial culture from blood or plural fluid except for coagulase negative *Staphylococcus spp*.
4. Paired sera with a fourfold or more increase in the titers of antibodies to *Mycoplasma pneumoniae* (MP), *Chlamydia pneumonia*, *L pneumophila or* respiratory viruses (Influenza A and B, Parainfluenza, Adenovirus, Respiratory syncytial virus)*.* Or Serum IgM antibody (MIF) ≥ 1:16 for *Chlamydia pneumonia.*

**Probable**, if one of the following criteria was met:

1. Detection of respiratory virus in sputum/bronchoalveolar lavage (BALF)/throat swabs by Realtime-PCR (Zhijiang, Shanghai, China) according to manufacturer’s instructions, including respiratory syncytial virus (RSV) types A and B, influenza virus (IFV) types A and B, parainfluenza virus (PIV) types 1, 2, 3 and 4, rhinovirus (HRV), enterovirus (EV), coronavirus (hCoV) types 229E, NL63, OC43 and HKU1, parapneumovirus (hMPV), and adenovirus (AdV), bocavirus;
2. Bacteria isolated form purulent sputum (defined as an adequate quality sputum sample with > 25 leukocytes and < 10 epithelial cells per × 100 magnification field) with compatible findings of Gram staining;
3. Detection of *Mycoplasma pneumoniae* (MP), *Chlamydia pneumonia* or *L pneumophila* in sputum/BALF/throat swabs by Real-time-PCR (Zhijiang, Shanghai, China)
4. Positive antigen for Influenza A/B (Alere ^TM^, Clearview Exact Influenza A& B)
5. Serum IgM antibody positive for *Mycoplasma pneumoniae* (MP), or Serum IgG antibody (MIF) ≥ 1:512 for *Chlamydia pneumonia.*

**Table S4. Recommendation of empirical therapy according to the 2016 Chinese CAP guideline**

| **Patients without risk factors of** [***Pseudomonas aeruginosa***](http://www.baidu.com/link?url=lLdomtoiR-Sm45dvsXYWt9YBtnpQNqL20JUaTiSfCb01AVA3uXkOJTwgMAA-WsNsjp-kh6UdHMfOUl2_xc_009bouXiFhxkLZAKDuUda0TI7I-VAuBDQ8mkkiC7dNdLW) **infection** | |
| --- | --- |
| **General ward patients** | 1. β-lactam^a^ (penicillin/inhibitor complex, third generation cephalosporins or inhibitor complex, cephamycins, oxacephems, ertapenem) 2. Macrolide + β-lactam^a^ 3. Respiratory fluoroquinolone |
| **ICU patients** | (1)Macrolide+β-lactam^b^ (penicillin/inhibitor complex, third generation cephalosporins or inhibitor complex, ertapenem)  (2) Respiratory fluoroquinolone + β-lactam^b^ |
| **Patients with risk factors of** [***Pseudomonas aeruginosa***](http://www.baidu.com/link?url=lLdomtoiR-Sm45dvsXYWt9YBtnpQNqL20JUaTiSfCb01AVA3uXkOJTwgMAA-WsNsjp-kh6UdHMfOUl2_xc_009bouXiFhxkLZAKDuUda0TI7I-VAuBDQ8mkkiC7dNdLW) **infection** | |
| **General ward patients** | 1. Antipseudomonal β-lactam^c^ 2. Antipseudomonal quinolone^d^ |
| **ICU patients** | 1. Antipseudomonal β-lactam^c^+ antipseudomonal quinolone 2. Antipseudomonal β-lactam^c^ + aminoglycoside 3. Antipseudomonal β-lactam^c^ + antipseudomonal quinolone+ aminoglycoside |

**^ab^**Penicillin/inhibitor complex= amoxicillin / clavulanic acid, amoxicillin / sulbactam, ampicillin / sulbactam

**^ab^**Third generation cephalosporins = ceftriaxone, cefotaxime, ceftizoxime, cefdinir, cefixime, cefpodoxime, cefditoren pivoxil

**^a^**Cephamycins=cefoxitin, cefminox, cefmetazole

**^a^**Oxacephems= moxalactam, flomoxef

**^c^**Antipseudomonal β-lactam = piperacillin/tazobactam, ticarcillin/clavulanic acid, mezlocillin/sulbactam, cefoperazone/sulbactam, ceftazidime, cefoperazone, cefepime, carbapenem (imipenem / cilastatin and meropenem)

**^d^**Antipseudomonal quinolone = levofloxacin, ciprofloxacin

**Table S5. Univariate analysis of prognostic factors of 60-day mortality in elderly CAP patients (n=3011)**

|  | **Survivors**  **(n=2787)** | **Non-survivors**  **(n=224)** | ***P* value** |
| --- | --- | --- | --- |
| Age (yr) | 77.17±7.34 | 80.57±7.74 | <0.001 |
| Male sex | 1510(54.2) | 130(58.0) | 0.282 |
| Long-term bedridden status | 203(7.3) | 56(25.0) | <0.001 |
| Aspiration | 255(9.1) | 58(25.9) | <0.001 |
| Underlying conditions |  |  |  |
| Cardiovascular disease | 1718(61.6) | 157(70.1) | 0.014 |
| Hypertension | 1354(48.6) | 123(54.9) | 0.072 |
| Ischemic heart disease | 879(31.5) | 91(40.6) | 0.005 |
| Congestive heart failure | 166(6.0) | 30(13.4) | <0.001 |
| Chronic respiratory disease | 793(28.5) | 48(21.4) | 0.026 |
| COPD | 519(18.6) | 40(17.9) | 0.782 |
| Bronchiectasis | 284(10.2) | 6(2.7) | 0.001 |
| Asthma | 148(5.3) | 7(3.1) | 0.161 |
| Dementia | 45(1.6) | 10(4.5) | 0.002 |
| Cerebral vascular disease | 692(24.8) | 90(40.2) | <0.001 |
| Chronic liver disease | 35(1.3) | 3(1.3) | 0.943 |
| Diabetes mellitus | 577(20.7) | 55(24.6) | 0.176 |
| Immunocompromise condition | 57(2.0) | 13(5.8) | <0.001 |
| Variables on admission |  |  |  |
| CURB-65 | 1.52±0.70 | 2.25±0.94 | <0.001 |
| WBC (10^9^·L^-1^) | 8.78±4.36 | 11.58±6.66 | <0.001 |
| Heart rate (beats·min^-1^) | 85.59±12.62 | 94.50±12.25 | <0.001 |
| Albumin (g·L^-1^) | 34.77±6.11 | 30.81±6.50 | <0.001 |
| Glucose (mmol·L^-1^) | 6.47±2.82 | 7.99±4.56 | <0.001 |
| Na^+^ (mmol·L^-1^) | 137.96±7.25 | 136.98±8.74 | 0.064 |
| SaO_2_ (%) | 93.91±9.56 | 90.50±12.25 | <0.001 |
| Multilobe infiltration | 1260(45.2) | 114(50.9) | 0.100 |
| Plural effusion | 662(23.8) | 87(38.8) | <0.001 |
| Antibiotic treatment |  |  |  |
| Adherent | 1081(38.8) | 66(29.5) | - |
| Undertreated | 463(16.6) | 71(31.7) | ＜0.001 |
| Overtreated | 1243(44.6) | 87(38.8) | 0.411 |

*Abbreviations*: COPD, chronic obstructive pulmonary disease; WBC, white blood cell; Na^+^, sodium; SaO_2_, arterial oxygen saturation.

*Note*: Loss to follow-up for patients was 62 cases. Data on empirical antimicrobial regimens in 49 patients were missing. 3 patients in general ward administrated antifungal agents and 6 patients in ICU administrated antipseudomonal β-lactam plus antifungal agents were ruled out.

**Table S6. Multivariable analysis of predictive factors of 60-day mortality in elderly CAP patients (n=3011)**

| **Predictive factors** | **OR** | **95%CI** | ***p* value** |
| --- | --- | --- | --- |
| Adherent to guideline or not | 0.8 | 0. 7-1.1 | 0.132 |
| Age (yr) | 1.04 | 1.01-1.07 | 0.013 |
| COPD | 1.2 | 0.3-4.5 | 0.778 |
| Asthma | 0.97 | 0.2-3.9 | 0.970 |
| Bronchiectasis | 0.4 | 0.1-1.4 | 0.135 |
| Congestive heart failure | 2.3 | 1.2-4.4 | 0.016 |
| Ischemic heart disease | 1.3 | 0.8-2.1 | 0.324 |
| Hypertension | 1.4 | 0.8-2.6 | 0.281 |
| Diabetes mellitus | 0.8 | 0.4-1.9 | 0.310 |
| Cerebral vascular disease | 0.9 | 0.6-1.5 | 0.737 |
| Dementia | 2.2 | 0.8-6.1 | 0.114 |
| Aspiration | 1.3 | 0.7-2.4 | 0.322 |
| Chronic liver disease | 0.9 | 0.2-4.8 | 0.899 |
| Immunocompromise condition | 2.3 | 0.8-7.1 | 0.136 |
| Long-term bedridden status | 2.1 | 1.2-3.7 | 0.010 |
| Heart rate (beats·min^-1^) | 1.01 | 1.01-1.03 | 0.005 |
| WBC (10^9^·L^-1^) | 1.04 | 0.99-1.08 | 0.072 |
| Albumin (g·L^-1^) | 0.9 | 0.90-0.97 | <0.001 |
| Glucose (mmol·L^-1^) | 1.06 | 1.00-1.12 | 0.044 |
| Na^+^ (mmol·L^-1^) | 0.99 | 0.98-1.02 | 0.871 |
| SaO_2_ (%) | 0.98 | 0.96-0.99 | 0.022 |
| Plural effusion | 1.4 | 0.9-2.1 | 0.111 |
| CURB65 | 2.0 | 1.6-2.5 | <0.001 |
| Multilobe infiltration | 1.1 | 0.7-1.6 | 0.785 |

*Abbreviations*: COPD, chronic obstructive pulmonary disease; WBC, white blood cell; Na^+^, sodium; SaO_2_, arterial oxygen saturation.

*Note*: Loss to follow-up for patients was 62 cases. Data on empirical antimicrobial regimens in 49 patients were missing. 3 patients in general ward administrated antifungal agents and 6 patients in ICU administrated antipseudomonal β-lactam plus antifungal agents were ruled out.
